# Supplementary material for: Identification of photoperiod-induced specific miRNAs in the adrenal glands of Sunite sheep (Ovis aries)
Source: Front Vet Sci. 2022 Jul 22;9:888207. doi: 10.3389/fvets.2022.888207 (PMC9354845; doi:10.3389/fvets.2022.888207)
Supplement: Supplementary file 1 [file Data_Sheet_1.ZIP › Supplementary file/miRDeep2 file.zip!/miRDeep2 file/Know_Structure/SP42A1/oar-mir-181a-2.pdf]

miRBase precursor : oar-mir-181a-2
Total read count : 17766
oar-miR-181a read count : 17612
remaining reads : 154

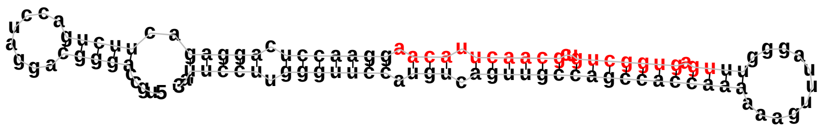

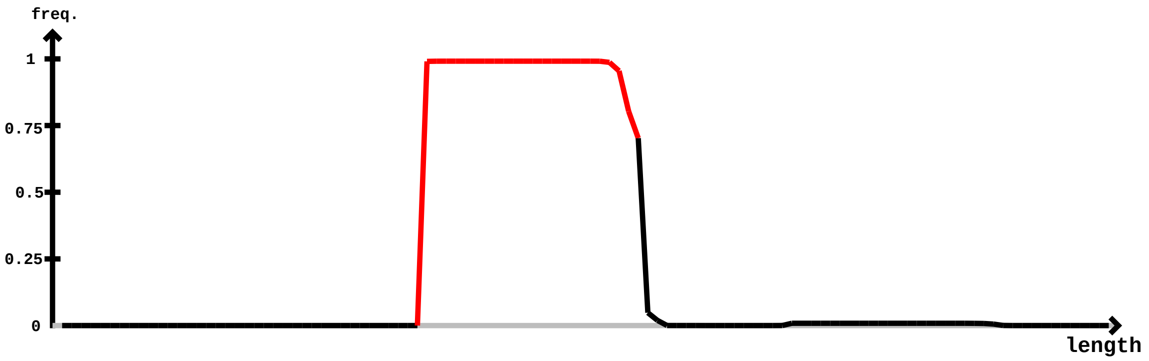


```
                                    oar-miR-181a
5'- ugccagggcaggauccagucuucagaggacuccaaggaacauucaacgcugucggugaguuugggauuugaaaaaaccaccgaccguugacuguaccuuggguuccuua   -3'    exp
    ....(((((........)))))..(((((.(((((((.(((.((((((..(((((((.((((...........))))))))))))))))).))).))))))).))))).   reads      mm          sample
    .....................................aacauucaacgcugucgg......................................................   3          0           seq
    .....................................aacauucaacgcugucggu.....................................................   77         0           seq
    .....................................aacauucaacgcugucggug....................................................   568        0           seq
    .....................................aacauucaacgcugucgguga...................................................   2694       0           seq
    .....................................aacauucaacgcugucggugag..................................................   1788       0           seq
    .....................................aacauucaacgcugucggugagu.................................................   11614      0           seq
    .....................................aacauucaacgcugucggugaguu................................................   511        0           seq
    .....................................aacauucaacgcugucggugaguuu...............................................   341        0           seq
    .....................................aacauucaacgcugucggugaguuug..............................................   2          0           seq
    .....................................aacauucaacgcugucggugaguuuggga...........................................   1          0           seq
    ......................................acauucaacgcugucggugagu.................................................   12         0           seq
    ......................................acauucaacgcugucggugaguu................................................   1          0           seq
    .......................................cauucaacgcugucggugaguuu...............................................   1          0           seq
    ..........................................................................aaccaccgaccguugacugu...............   1          0           seq
    ..........................................................................aaccaccgaccguugacuguac.............   3          0           seq
    ...........................................................................accaccgaccguugacugu...............   4          0           seq
    ...........................................................................accaccgaccguugacugua..............   9          0           seq
    ...........................................................................accaccgaccguugacuguac.............   39         0           seq
    ...........................................................................accaccgaccguugacuguacc............   90         0           seq
    ...........................................................................accaccgaccguugacuguaccu...........   7          0           seq
```
